# Begelomab (BEGESAND^®^) Salvages Steroid-Resistant Acute GVHD in Pediatric Patients

**DOI:** 10.3390/jcm15114190

**Published:** 2026-05-28

**Authors:** David Shyr, Steven M. Chirieleison, Sebastian Fernandez-Pol, Katja Weinacht, Rajni Agarwal, Ami J. Shah, Michela Spinelli, Renata Palmieri, Antonio Francesco Di Naro, Alice Bertaina

**Affiliations:** 1 School of Medicine, Stanford University, Stanford, CA 94305, USA; schiriel@stanford.edu (S.M.C.); sfernand@stanford.edu (S.F.-P.); kgw1@stanford.edu (K.W.); rajnia@stanford.edu (R.A.); ashah5@stanford.edu (A.J.S.); aliceb1@stanford.edu (A.B.); 2 Adienne Pharma & Biotech, 6900 Lugano, Switzerland; michela.spinelli@adienne.com (M.S.); renata.palmieri@adienne.com (R.P.); afdinaro@adienne.com (A.F.D.N.)

**Keywords:** Begelomab, graft-versus-host disease, stem cell transplant

## Abstract

**Background**: Acute graft-versus-host disease (aGVHD) is a leading cause of morbidity and mortality following pediatric hematopoietic stem cell transplantation (HSCT). Approximately half of children achieve complete response (CR) to corticosteroids, whereas steroid-refractory (SR) disease carries a 1–2-year mortality of 41–44%. Mortality risk is 2.6-fold higher in children > 13.9 years, and respiratory failure accounts for 26% of deaths. Existing second-line agents—ruxolitinib, tocilizumab, or extracorporeal photopheresis—have delayed onset or high toxicity. Begelomab (BEGESAND^®^), a monoclonal antibody targeting CD26/dipeptidyl peptidase-4 (DPP4), inhibits CD26-mediated T-cell activation and has demonstrated 75% response in adults with minimal toxicity. However, pediatric data are lacking. **Methods**: We retrospectively reviewed five consecutive pediatric patients (ages 3–20 years) treated with Begelomab (BEGESAND^®^) for SR (*n* = 4) or steroid-dependent (SD; *n* = 1) aGVHD between 2017–2021 under emergency IND authorization. Begelomab (BEGESAND^®^) was administered intravenously at 2.7 mg/m^2^/day on days 1–5, 10, 14, 17, 21, 24, and 28. GVHD was graded by MAGIC criteria; flow cytometry and immunohistochemistry (IHC) assessed CD26 expression and immune effects. **Results**: All patients had grade IV disease after ≥2 prior agents. Two with pre-existing sepsis died early, before treatment response could be assessed. Of three evaluable patients, two (67%) achieved CR within 21 days and one achieved durable control by 6 months. All three remain alive; no Begelomab (BEGESAND^®^)-related toxicity, cytopenia, or new infections occurred. Flow cytometry showed preserved T-cell subsets, and IHC demonstrated CD26 localization at sites of epithelial injury. **Conclusions**: Begelomab (BEGESAND^®^) showed promising timely and durable responses with excellent safety in pediatric SR/SD-aGVHD, supporting further evaluation in multicenter pediatric trials.

## 1. Introduction

Graft-versus-host disease (GVHD) remains one of the major competing causes for transplant-related mortality in HSCT after disease relapse [1,2]. Although approximately 50% of pediatric patients achieve complete response (CR) within four weeks of initiating glucocorticoid therapy, transplant-related mortality (TRM) is significantly elevated in steroid-refractory cases [3]. The prognosis for pediatric patients with steroid-refractory (SR) acute GVHD remains poor, with overall mortality approaching 41% at one year and 44% at two years [4]. Only 30% of children with SR-GVHD achieve disease resolution by day 100 post-HSCT [4]. Mortality risk is further elevated in older children (>13.9 years), who experience 2.6-fold higher death rates compared to younger patients. Respiratory insufficiency from progressive GVHD accounts for approximately 26% of deaths in this population [4]. These sobering outcomes underscore the urgent need for effective, well-tolerated second-line therapies. The recent harmonization of steroid-refractory (SR) and steroid-dependent (SD) GVHD definitions by the EBMT-NIH-CIBMTR Task Force enables the earlier initiation of second-line therapies [5]. However, the management of SR/SD GVHD continues to be a major clinical challenge in the pediatric population, where unique physiologic factors limit the use of many established agents [6,7]. Oral medications are difficult to administer and demonstrate variable gastrointestinal absorption [8]. Ruxolitinib, the main choice of second-line agents, exhibits reduced clearance in GVHD patients, often resulting in myelosuppression, treatment interruptions, and heightened risks of infection and bleeding [9,10]. Extracorporeal photopheresis (ECP), another major second-line option, requires central vascular access—often challenging in children and associated with increased risk of apheresis-related complications [11,12]. Additionally, biologics such as vedolizumab and tocilizumab may take weeks to achieve therapeutic efficacy [13,14]. The recent FDA approval of remestemcel-L for aGVHD in pediatrics underscores the clinical need for IV agents with favorable toxicity profiles and a rapid onset of action in the pediatric setting [15].

Begelomab (BEGESAND^®^) is a murine monoclonal antibody targeting human dipeptidyl peptidase 4 (DPP4/CD26), inhibiting CD26-mediated T-cell activation in various tissues [16]. Previous studies have demonstrated that CD26 enzymatic activity modulates chemokines and cytokines, thereby influencing immune cell trafficking and inflammatory responses, providing a rationale for targeting CD26 to reduce T-cell-mediated inflammation [17]. In addition, increased CD26 expression on donor stem cells and post-transplant immune cells, as well as elevated soluble CD26 levels, has been correlated with a higher risk of graft-versus-host disease (GVHD), supporting CD26 as a predictive biomarker and therapeutic target in allogeneic HSCT [18,19]. DPP4 inhibition with small-molecule inhibitors such as sitagliptin, in combination with tacrolimus and sirolimus, has reduced the incidence of acute GVHD in clinical trials, further supporting the clinical applicability of CD26/DPP4 blockades in this setting [20,21]. Together, these data provide a strong mechanistic and clinical rationale for evaluating Begelomab (BEGESAND^®^), a monoclonal antibody targeting CD26/DPP4, as an antibody-based strategy for the treatment of steroid-refractory and steroid-dependent aGVHD, particularly in high-risk pediatric patients. Begelomab (BEGESAND^®^) has received orphan drug designation by the FDA and EMA for the treatment of GvHD. A European study of BEGESAND^®^ in adult patients with grade ≥ 2 acute GVHD (aGVHD) demonstrated a 75% overall response rate by day 28, including 83% response in grade III and 66% response in grade IV aGVHD [22]. Notably, no Begelomab (BEGESAND^®^)-related adverse events were reported. Given these findings, Begelomab (BEGESAND^®^) represents a potential solution as a rapidly acting intravenous agent with minimal toxicity, addressing a critical unmet need in pediatric SR-GVHD. To date, no published data exist on Begelomab (BEGESAND^®^) use in children. We present the first pediatric experience using Begelomab (BEGESAND^®^) for SR aGVHD at a single institution. Preliminary findings from this pediatric Begelomab (BEGESAND^®^) experience were previously presented in abstract form at the 2024 American Society for Transplantation and Cellular Therapy (ASTCT) Annual Meeting, and the current report provides the first comprehensive, peer-reviewed description of these cases [23].

## 2. Materials and Methods

### 2.1. Study Design

This study was a retrospective review of five consecutive pediatric patients treated with Begelomab (BEGESAND^®^) for SR aGVHD between 2017 and 2021. The patients received BEGESAND^®^ as an emergency investigational new drug (eIND), approved by the FDA (eIND number 137878, 142201, 142738, 147193, 156645). Begelomab (BEGESAND^®^) was administered intravenously at 2.7 mg/m^2^/day on days 1, 2, 3, 4, 5, 10, 14, 17, 21, 24, and 28 (a total of 11 doses) under emergency IND (eIND) authorization.

GVHD diagnosis, staging, and grading were determined according to the Mount Sinai Acute GVHD International Consortium (MAGIC) criteria [24]. Clinical data were extracted from electronic medical records by two independent investigators and verified by a third. Variables collected included patient demographics, underlying disease, donor type and HLA match, graft source, conditioning regimen, GVHD prophylaxis, day of GVHD onset, maximum GVHD grade before treatment, interval between GVHD diagnosis and Begelomab (BEGESAND^®^) initiation, concurrent GVHD therapies, complications at time of treatment, days to best response, and post-treatment GVHD grade. Complete response was defined as the complete resolution of symptoms attributable to GVHD. Partial response was defined as a decrease in GVHD grade according to MAGIC criteria. Steroid-refractory or -resistant aGVHD was defined as (1) progression in any organ within 5 days of therapy onset with ≥2 mg/kg/day of prednisone equivalent, (2) failure to improve within 7 days of treatment initiation, or (3) incomplete response after more than 28 days of immunosuppressive treatment including steroids. Steroid dependence was defined as the inability to taper prednisone under 2 mg/kg/day after an initial response of at least 7 days or as recurrence of aGvHD activity during steroid taper. When feasible, multicolor flow cytometry and histopathological studies were performed before and after Begelomab (BEGESAND^®^) treatment to assess immunophenotype and CD26 expression in affected tissues.

### 2.2. Flow Cytometry Immunophenotyping

Flow cytometry was performed to evaluate: (1) CD26 expression levels on T-cell subsets pre- and post-treatment to assess antibody-mediated downregulation or depletion; and (2) T-cell subset distribution (CD4+, CD8+, regulatory T-cells) to characterize immune reconstitution to monitor treatment response. Peripheral blood was analyzed using a 10-color Lyric flow cytometer (BD Biosciences, San Jose, CA, USA). A validated T-cell immunotherapy panel included: CD8-FITC, CD26-PE, CD4 PerCP-Cy5.5, CD52 PE-Cy7, CD30 APC, CD3 APC-H7, CCR4 BV421, CD45 V500, CD25 APC-R700, CD7 BV605, and CD19 PE-Cy7. Data were analyzed with FCS Express 7 Software, De Novo Software, Pasadena, CA, USA, version, with a minimum of 200,000 events acquired per sample.

### 2.3. Immunohistochemical Staining of CD26

CD26 expression was assessed using a rabbit monoclonal antibody (Cell Signaling Technology #40134, Danvers, MA, USA). Formalin-fixed paraffin-embedded tissues were sectioned at 5 µm and stained per the manufacturer’s protocol. Slides underwent deparaffinization, pH 6 citrate antigen retrieval, blocking, and overnight primary antibody incubation (1:200 dilution). Detection was performed using HRP-conjugated secondary antibodies and DAB chromogen. Slides were mounted in DPX for microscopic examination.

## 3. Results

### 3.1. Patient Characteristics and Treatment

Five pediatric patients (ages 3–20 years) received Begelomab (BEGESAND^®^) for steroid-refractory (*n* = 4) or steroid-dependent (*n* = 1) aGVHD between 2017 and 2021 (Table 1). Four patients had undergone allogeneic HSCT for acute leukemia. One patient with end-stage renal disease secondary to focal segmental glomerulosclerosis received a haploidentical HSCT from the same donor who provided a simultaneous kidney transplant.

Acute GVHD developed between post-transplant days 10 and 32 (median day 21). All patients presented with grade IV disease involving at least one organ at stage IV severity: gut alone (*n* = 2), gut plus liver (*n* = 1), skin alone (*n* = 1), or skin plus liver (*n* = 1). All patients were heavily pre-treated with methylprednisolone 2 mg/kg/day plus a median of 3 additional second-line agents (range 2–5) prior to Begelomab Begelomab (BEGESAND^®^) initiation. These agents included calcineurin inhibitors (*n* = 3 patients given TCR alpha beta depleted HSCT and who were not on pharmacological prophylaxis), infliximab (*n* = 4), ruxolitinib (*n* = 3), extracorporeal photopheresis (*n* = 2), basiliximab (*n* = 2), alpha-1-antitrypsin (*n* = 2), and intra-arterial corticosteroids for refractory gastrointestinal GVHD (*n* = 2). The interval from initial GVHD diagnosis to Begelomab (BEGESAND^®^) initiation ranged from 28 to 112 days (median 63 days), reflecting delays inherent to compassionate use access.

Begelomab (BEGESAND^®^) was administered at 2.7 mg/m^2^/day following the standard regimen (days 1–5, 10, 14, 17, 21, 24, and 28). Three patients completed all 11 doses. Two patients with pre-existing sepsis and multiorgan failure received 7 and 10 doses, respectively, before succumbing to transplant-related complications.

### 3.2. Clinical Outcomes

Two patients died before GVHD response to Begelomab (BEGESAND^®^) could be assessed. Patient 2 died on day 89 post-transplant after 10 doses of Begelomab (BEGESAND^®^) from complications of pre-existing polymicrobial sepsis and progressive multiorgan failure. Patient 3 died on day 149 post-transplant after 7 doses from refractory septic shock and respiratory failure. Both deaths were attributed to infectious complications predating Begelomab (BEGESAND^®^) initiation rather than treatment-related toxicity.

Among the three evaluable patients, two (Patients 1 and 4) achieved a complete resolution of GVHD symptoms within 21 days of starting Begelomab (BEGESAND^®^) and were successfully weaned off systemic corticosteroids within 6 weeks. Patient 5, who had steroid-dependent GVHD with recurrent flares during taper attempts, achieved sustained disease control and was weaned off all immunosuppression except low-dose tacrolimus by 6 months post-treatment. At last follow-up (range 8–36 months), all three responding patients remained alive without GVHD recurrence.

### 3.3. Safety and Tolerability

No adverse events were attributable to Begelomab (BEGESAND)^®^. Notably, Begelomab (BEGESAND)^®^ administration did not appear to worsen pre-existing infections in the two patients with active sepsis at treatment initiation. No new opportunistic infections, cytopenias, or infusion reactions were observed during or after the treatment course.

### 3.4. Immunophenotypic and Tissue Analysis

Immunophenotypic and tissue analyses were limited by the compassionate-use context and critical illness of several patients; CD26 immunohistochemistry was available for three patients (Patients 1–3), and longitudinal flow cytometry was performed in one patient (Patient 5).

**Figure 1:**
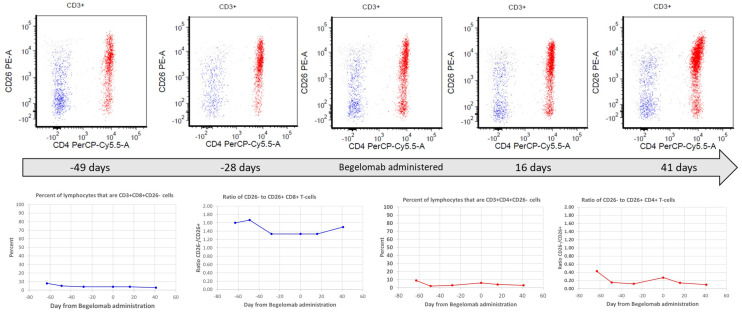
Expression of CD26 as assessed by peripheral blood flow cytometry in Patient 5. Red population represents CD4+ T-cells. Blue population represents CD8+ T = cells. Light gray population represents primarily CD4/8 double negative T-cells. There was a mild downtrend in the percentage of lymphocytes that were CD3+CD4+CD26- and CD3+CD8+CD26- after begelomab within physiological variability.

Peripheral blood flow cytometry performed in Patient 5 before and after completing Begelomab (BEGESAND)^®^ therapy revealed no depletion of CD26-expressing lymphocytes and no downregulation of CD26 surface expression on CD4+ or CD8+ T-cell subsets (Figure 1). Absolute lymphocyte counts and T-cell subset distributions remained stable throughout treatment; although the CD4:CD8 ratio increased from 3.7 at Begelomab (BEGESAND)^®^ initiation to 5.9 on day 41, a causal relationship to Begelomab (BEGESAND)^®^ is uncertain.

**Figure 2:**
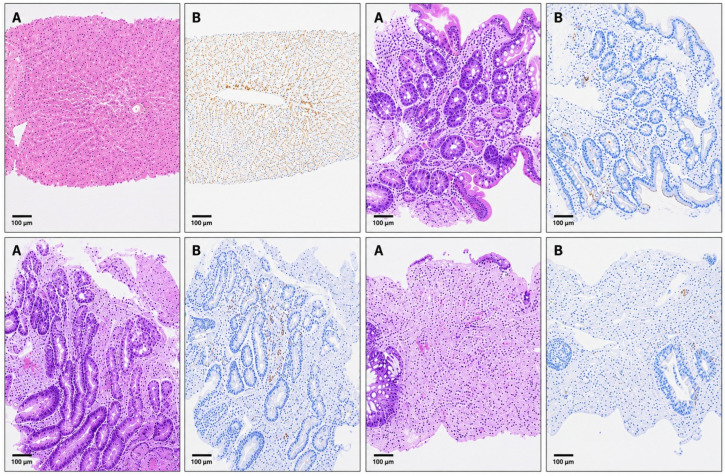
(**left-top**) Liver biopsy from Patient 1 showing pericentral area. (A) H&E image of peri-central liver core with cholestasis and associated hepatocyte injury. (B) CD26 immunohistochemistry demonstrating canalicular hepatocyte staining in a zonal distribution. Predominant staining in zone 3 extending to zone 2 and sparing zone 1; (**right-top**) Duodenum biopsy from Patient 3 demonstrating grade 1 GVHD. (A) H&E image demonstrating increased crypt apoptosis compatible with grade 1 GVHD. There is no crypt dropout, ulceration, or lamina propria fibrosis. (B) Immunohistochemical stain for CD26 demonstrating luminal brush border staining as well as cytoplasmic and membranous staining of crypt epithelium, more pronounced in withering crypts.; (**left-bottom**) Stomach biopsy from Patient 2 with grade 1 GVHD (A) H&E of stomach biopsy demonstrating increased crypt apoptosis with associated ballooning degeneration. (B) CD26 immunohistochemical stain showing CD26 staining in a cytoplasmic and membranous pattern of scattered withering crypts.; (**right-bottom**) Rectum biopsy from Patient 3 with grade 4 GVHD (A) H&E of rectal biopsy demonstrating abundant crypt apoptosis with associated crypt drop out and lamina propria fibrosis. (B) CD26 immunohistochemical stain showing luminal CD26 staining in addition to cytoplasmic and membranous staining of scattered crypt epithelial cells with preferential staining in withered crypts.

Immunohistochemical analysis of tissue biopsies from GVHD-affected organs demonstrated CD26 expression at sites of active disease. In liver biopsies from Patient 1, CD26 staining showed granular membranous and cytoplasmic localization along hepatocyte canalicular surfaces, consistent with the known expression pattern in biliary epithelium. In gastrointestinal biopsies (Patient 2—stomach and Patient 3—duodenum/rectum), CD26 staining was prominently concentrated within withering intestinal crypts—hallmark sites of apoptotic injury in GVHD—with minimal staining in intact crypt architecture (Figure 2). This pattern supports the localization of CD26 at sites of active epithelial injury in GVHD-affected tissues (Figure 2). This pattern suggests preferential CD26 expression or antibody accessibility at sites of active epithelial damage.

## 4. Discussion

This case series represents the first reported pediatric experience with Begelomab (BEGESAND)^®^ for SR/SD acute GVHD. Despite the small cohort and heavily pre-treated patient population, Begelomab (BEGESAND)^®^ demonstrated encouraging clinical activity: among three evaluable patients, two achieved a complete resolution of grade IV GVHD within 21 days, and one achieved sustained immunosuppression taper by 6 months. These response rates compare favorably to published pediatric outcomes with other second-line agents, where overall response rates range from 30–60% and mortality approaches 41–44% at 1–2 years. Notably, both patients achieving complete response had gut involvement, while the patient with skin-predominant GVHD achieved sustained disease control with ongoing tacrolimus. This pattern suggests potential organ-specific efficacy, consistent with high CD26 expression in intestinal epithelium. Whether Begelomab (BEGESAND)^®^ demonstrates preferential activity against gastrointestinal GVHD—the organ system associated with the highest mortality—warrants systematic evaluation in future studies. Our pediatric findings align with the European adult experience reported by Bacigalupo et al., where Begelomab (BEGESAND)^®^ achieved 75% overall response rates, with a 83% response in grade III and a 66% response in grade IV aGVHD. The comparable efficacy, despite our patients receiving Begelomab (BEGESAND)^®^ as a later-line therapy (median 3 prior agents) and having 100% grade IV disease, suggests robust activity across age groups. However, pediatric patients may benefit from more rapid immune reconstitution post-HSCT, potentially enhancing their response to immunomodulatory agents. Direct age-stratified comparisons in prospective trials are needed to confirm these observations.

The rapid onset of clinical benefit—evident within 3 weeks in responding patients—represents a key advantage in pediatric SR-GVHD, where delayed therapeutic efficacy compounds the risk of progressive organ damage and opportunistic infections. In comparison, biologics such as vedolizumab and tocilizumab typically require 4–8 weeks to achieve full therapeutic effect. This rapid response, combined with the ease of intravenous administration and favorable safety profile, positions Begelomab (BEGESAND)^®^ as a potentially valuable option in critically ill children where oral absorption is unreliable and vascular access for extracorporeal photopheresis is challenging.

A notable feature of Begelomab (BEGESAND)^®^ is its favorable safety profile. No treatment-related adverse events were observed, including in two patients with active sepsis at treatment initiation, suggesting that Begelomab (BEGESAND)^®^ does not significantly impair antimicrobial immunity. Flow cytometry analysis demonstrated that Begelomab (BEGESAND^®^) did not appear to deplete CD26-expressing T-cells or downregulate surface CD26 expression, suggesting a potentially non-depleting mechanism of action. This observation raises the possibility that Begelomab may exert its therapeutic effects through mechanisms distinct from lymphocyte-depleting agents such as anti-thymocyte globulin and alemtuzumab, which are associated with substantial risks of prolonged immunosuppression, viral reactivation, and secondary malignancies. The preservation of T-cell populations may also help maintain graft-versus-leukemia activity, an important consideration in patients undergoing transplantation for malignant diseases. Given these preliminary findings, longitudinal flow cytometric assessments of CD26 expression should be incorporated into future clinical trials of Begelomab (BEGESAND^®^) to validate and further characterize this observation.

The mechanism by which Begelomab (BEGESAND)^®^ exerts its therapeutic benefit on GVHD remains incompletely understood. CD26/DPP4 is a multifunctional serine protease expressed on activated T-cells, where it serves as a costimulatory molecule and regulator of chemokine activity in different tissues, including the gut [19,20,25]. 

Begelomab (BEGESAND)^®^’s efficacy without T-cell depletion suggests a functional blockade of CD26-mediated T-cell activation pathways, potentially inhibiting alloreactive T-cell proliferation and tissue infiltration [26]. Our immunohistochemical findings provide additional mechanistic insight: CD26 expression was prominently detected in GVHD-affected tissues, particularly within withering intestinal crypts and along hepatocyte canalicular surfaces—hallmark sites of epithelial apoptosis in GVHD. This localization pattern suggests that Begelomab (BEGESAND)^®^ may access and exert activity at sites of active tissue injury, consistent with prior observations in dermatomyositis where Begelomab (BEGESAND)^®^ demonstrated efficacy in another inflammatory tissue-destructive disease [16]. Whether CD26 expression in damaged epithelium represents a disease biomarker or therapeutic target warrants further investigation. Evaluating soluble CD26 levels and CD26 enzymatic activity in plasma at defined time points in future studies would also provide important insight into the mechanism of action of Begelomab (BEGESAND)^®^, help clarify the relationship between tissue CD26 expression and systemic DPP4 activity, and may identify biomarkers to guide its use in GVHD and other immune-mediated diseases.

A critical limitation of this experience is the delayed treatment initiation, with a median of 63 days from aGVHD diagnosis to Begelomab (BEGESAND)^®^ administration (range 28–112 days). This delay reflects the constraints of compassionate use access and the necessity of exhausting multiple second-line therapies before obtaining emergency IND approval. Earlier intervention may improve outcomes, as tissue damage becomes progressively irreversible with prolonged GVHD activity [27,28]. The poor outcomes in two patients with established multiorgan failure underscore the importance of timely escalation. Prospective evaluation should prioritize Begelomab (BEGESAND)^®^ administration within 14–28 days of steroid refractoriness to optimize the therapeutic window. Additional limitations include the small sample size, retrospective design, heterogeneous prior therapies, and absence of standardized immune monitoring. The inclusion of one patient receiving HSCT for immune tolerance induction rather than malignancy adds biological heterogeneity, although this patient’s response demonstrates Begelomab (BEGESAND)^®^’s potential applicability beyond oncologic indications. Formal pharmacokinetic studies in children are needed, as pediatric drug disposition may differ from adult populations. Finally, longer follow-up is required to assess the durability of responses, chronic GVHD incidence, and relapse rates in the leukemia patients.

Despite these limitations, the rapid responses, favorable safety profile, and ease of administration observed in this pediatric cohort support further investigation of Begelomab (BEGESAND)^®^ for SR/SD GVHD. The non-depleting mechanism, lack of infectious complications even in septic patients, and potential for early steroid sparing represent clinically meaningful advantages in a vulnerable population with limited therapeutic options. Compared with commonly used second-line agents such as ruxolitinib, vedolizumab, and tocilizumab—which may be limited by myelosuppression, infectious risk, logistical complexity, or delayed onset of action—Begelomab (BEGESAND)^®^ provided rapid responses, an intravenous route of administration, and no apparent additional toxicity in this pediatric series, although definitive comparative efficacy and safety cannot be established from this small, retrospective cohort. Prospective multicenter trials are warranted to define optimal dosing, timing, patient selection criteria, and comparative efficacy against established second-line agents. Correlative studies evaluating CD26 expression patterns, circulating DPP4 enzyme activity, and T-cell functional assays may identify predictive biomarkers to guide patient selection and monitoring. International collaboration and pediatric-specific trial design will be essential to fully elucidate Begelomab (BEGESAND)^®^’s role in the treatment algorithm for pediatric acute GVHD.

## Figures and Tables

**Table 1:** Patient, Transplant and GVHD Characteristics.

Characteristic	Patient 1	Patient 2	Patient 3	Patient 4	Patient 5
Age (years)	3	18	14	11	20
Sex	M	M	M	M	M
Diagnosis	AML	AML	ALL	AML	FSGS
Donor/HLA Match	MMUD 9/10	MMRD 5/10	MMRD 6/10	MUD 10/10	MMRD 5/10
Graft Source	BM	PBSC	PBSC	BM	PBSC
Conditioning	ATG/Bu/Cy	ATG/Bu/Mel	ATG/TBI/TT/Flu	ATG/Bu/Cy/Mel	ATG/Flu/Cy/Mel
GVHD Prophylaxis	CSA/MTX	TCRαβ/CD19 depletion	TCRαβ/CD19 depletion	CSA/MTX	TCRαβ/CD19 depletion
GVHD Dx (Post-Tx Day; Biopsy Before Begelomab)	12; liver	23; GI	10; GI	21; GI	32; no biopsy
GVHD Stage Before Begelomab	S0, G4, L2/Gr4	S0, G4, L0/Gr4	S1, G4, L4/Gr4	S4, G0, L0/Gr4	S0, G0, L0/Gr0 (max S4, L2/Gr4)
Days from Dx → Begelomab	104	112	54	28	63
Total Dose	11	7	10	11	11
Concomitant Treatment	Infliximab; ECP	Infliximab; IA steroids	Infliximab; basiliximab; ruxolitinib	Ruxolitinib	Tacrolimus; A1PI; basiliximab; ruxolitinib
Complications	VOD; adenovirus	ARDS; AKI; CMV; BK	VOD; bacteremia; CMV; BK; RSV	Hemorrhagic cystitis; CMV	None
Days to Response	14	NA	NA	21	174
Post-GVHD Stage	S0, G0, L0/Gr0	NA	NA	S0, G0, L0/Gr0	S0, G0, L0/Gr0
Outcome	CR	Death	Death	CR	CR

ARDS—acute respiratory distress syndrome, AKI—acute renal injury, AML—acute myeloid leukemia, ATG—thymoglobulin, BM—bone marrow, Bu—busulfan, CONs—coagulase negative staphylococcus, CMV—cytomegalovirus, CR—complete response, CSA—cyclosporine, Cy—cyclophosphamide, ECP—extracorporeal phostopheresis, FSGS—focal sclerosing glomerulonephritis, G—gut, Gr—grade, GVHD—graft versus host disease, HLA—human leukocyte antigen, L—liver, Mel—melphalan, MMRD—mismatched related (haploidentical), MMUD—mismatched unrelated, MTX, methotrexate, MUD—matched unrelated, PBSC—peripheral blood stem cell, RSV—respiratory syncytial virus, S—skin, TCRαβ—T-cell receptor alpha beta, Tx—transplant, VOD—veno—occlusive disease.

## Data Availability

The data underlying this study are not publicly available due to patient privacy and institutional restrictions but are available from the corresponding author on reasonable request and with permission from the institutional review board.
